# Supplementary material for: Research Progress on the Species and Diversity of Ants and Their Three Tropisms
Source: Insects. 2023 Nov 18;14(11):892. doi: 10.3390/insects14110892 (PMC10672356; doi:10.3390/insects14110892)
Supplement: Supplementary file 1 [file insects-14-00892-s001.zip › insects-2660410-supplementary.pdf]

Table S1 List of subfamilies and genus, and the number of respective species of ants in China

Subfamily	Genus	Species
Amblyoponinae (Total 4 genera, 13 species)	*Amblyopone* (10 species)	*Amblyopone actodentata*
		Amblyopone bruni
		Amblyopone crenata
		Amblyopone eminia
		Amblyopone rothneyi
		Amblyopone rubiginoa
		Amblyopone sakaii
		Amblyopone silvestrii
		Amblyopone triloba
		Amblyopone zaojun
	Bannapone (1 species)	*Bannapone mulanae*
	Mystrium (1 species)	*Mystrium coulatum*
	Prionopelta (1 species)	*Prionopelta kraepelini*
Dolichoderinae (Total 7 genera, 30 species)	*Bothriomyrmex* (1 species)	*Bothriomyrmex kusnezovi*
	Chronoxenus (1 species)	*Chronoxenus dalyi*
	Dolichoderus (15 species)	*Dolichoderus affinis*
		Dolichoderus breviscapus
		Dolichoderus brunnus
		Dolichoderus dajiensis
		Dolichoderus feae
		Dolichoderus flatidorsus
		Dolichoderus flavus
		Dolichoderus incisus
		Dolichoderus pilosus
		Dolichoderus roticepus
		Dolichoderus rugocapitus
		Dolichoderus sagmanotus
		Dolichoderus sibiricus
		Dolichoderus squamanodus
		Dolichoderus taprobanae
	Iridomyrmex (1 species)	*Iridomyrmex anceps*
	Liometopum (2 species)	*Liometopum lindgreeni*
		Liometopum sinense
	Tapinoma (6 species)	*Tapinoma geei*
		Tapinoma melanocephalum
		Tapinoma orthocephalum
		Tapinoma rectinotum
		Tapinoma simrothi
		Tapinoma sinense

	Technomyrmex (4 species)	*Technomyrmex albipes*
		Technomyrmex antennus
		Technomyrmex bicolor
		Technomyrmex brunneus
Ectatomminae (Total 1 genus, 7 species)	*Gnamptogenys* (7 species)	*Gnamptogenys bicolor*
		Gnambtogenys binghami
		Gnamptogenys coccina
		Gnamptogenys panda
		Gnamptogenys sichuanensis
		Gnamptogenys sinensis
		Gnamptogenys taicanensis
Formicinae (Total 20 genera, 294 species)	*Acropyga* (6 species)	*Acropyga acuirentris*
		Acropyga butteli
		Acropyga nipponensis
		Acropyga sauteri
		Acropyga yaeyamensis
		Acropygra yashi
	Anoplolepis (1 species)	*Anoplolepis gracilipes*
	Camponotus (86 species)	*Camponotus aethiops*
		Camponotus albivillosus
		Camponotus albosparsus
		Camponotus anningensis
		Camponotus atrox
		Camponotus auratiacus
		Camponotus badius
		Camponotus barbatus
		Camponotus bedoti
		Camponotus bonariensis
		Camponotus breriscapus
		Camponotus carin
		Camponotus carin subsp.tipuna
		Camponotus chongqingensis
		Camponotus compressus
		Camponotus confucii
		Camponotus cornis
		Camponotus derestirus
		Camponotus dolendus
		Camponotus exiguoguttatus
		Camponotus fedaschenkoi
		Camponotus fextinus
		Camponotus formosensis
		Camponotus friedae
		Camponotus fuscirillosus

		Camponotus guizhouensis
		Camponotus habereri
		Camponotus helous
		Camponotus herculeanus
		Camponotus holosericeus
		Camponotus holthoffi
		Camponotus humerus
		Camponotus interjectus
		Camponotus irritans
		Camponotus irritans subsp.honghongensis
		Camponotus itoi
		Camponotus japonicus
		Camponotus jianghuaensis
		Camponotus kiusiuensis
		Camponotus kurdistanicus
		Camponotus laotzei
		Camponotus largiceps
		Camponotus lasiselene
		Camponotus leonardi
		Camponotus liehti
		Camponotus ligniperdus
		Camponotus longiceps
		Camponotus minus
		Camponotus mitis
		Camponotus monju
		Camponotus nicobarensis
		Camponotus nipponicus
		Camponotus obscuripes
		Camponotus parius
		Camponotus piceus
		Camponotus politae
		Camponotus pseudoirritans
		Camponotus pseudolendus
		Camponotus punctatissimus
		Camponotus quadrinotatus
		Camponotus ragus
		Camponotus ranispinus
		Camponotus rariegatus
		Camponotus rariegatus subsp. proles
		Camponotus reichardti
		Camponotus ritreus

		Camponotus rothneyi subsp. tairanae
		Camponotus rubidus
		Camponotus rufoglaucus
		Camponotus sachalinensis
		Camponotus saxatilis
		Camponotus selene
		Camponotus semirafus
		Camponotus siemsseni
		Camponotus singularis
		Camponotus spanis
		Camponotus toi subsp. facansienensis
		Camponotus tokioensis
		Camponotus tonkinus
		Camponotus truebi
		Camponotus turkestanicus
		Camponotus turkestanus
		Camponotus variegatus
		Camponotus wasmanni
		Camponotus xingdoushanensis
		Camponotus yiningensis
	Cataglyphis (7 species)	*Cataglyphis aenescens*
		Cataglyphis emeryi
		Cataglyphis flaviribia
		Cataglyphis glabilabia
		Cataglyphis helanensis
		Cataglyphis italicus
		Cataglyphis pallidus
	Formica (45 species)	*Formica altayensis*
		Formica approrimans
		Formica aquilonia
		Formica aseta
		Formica beiiineensis
		Formica breviscapa
		Formica candida
		Formica candida subsp.formosae
		Formica changhei
		Formica cinerea
		Formica cinereo
		Formica clara
		Formica cunicularia
		Formica dachaidanensis

		Formica delinghaensis
		Formica eongi
		Formica ersecta
		Formica fukaii
		Formica fusca
		Formica gagates
		Formica gagatoides
		Formica glabridorsis
		Formica iabonica
		Formica lemani
		Formica liogaster
		Formica liopthalma
		Formica lugubris
		Formica mesasiatica
		Formica minioccaChang
		Formica obsidiana
		Formica pamirica
		Formica polyctena
		Formica pratensis
		Formica rufa
		Formica rufibarbis
		Formica sanguinea
		Formica sentschuensis
		Formica sinae
		Formica sinensis
		Formica subpilosa
		Formica subrufa
		Formica truncorum
		Formica uralensis
		Formica villiscapa
		Formica yessensis
	Gesomyrmex (1 species)	*Gesomyrmer howardi*
	Lasius (19 species)	*Lasius alienus*
		Lasius capitatus
		Lasius carniolicus
		Lasius coloratus
		Lasius emarginatus
		Lasius flavus
		Lasius fuji
		Lasius fuliginosus
		Lasius hayashi
		Lasius himalayanus
		Lasius japonicus

		Lasius longicirrus
		Lasius myops
		Lasius niger
		Lasius nipponensis
		Lasius obscuratus
		Lasius productus
		Lasius talpa
		Lasius umbratus
	Lepisiota (8 species)	*Lepisiota acuta*
		Lepisiota capensis
		Lepisiota heriangu
		Lepisiota opaca
		Lepisiota pulchella
		Lepisiota reticulata
		Lepisiota rothneyi
		Lepisiota xichangensis
	Myrmoteras (2 species)	*Myrmoteras binghamii*
		Myrmoteras cuneonodum
	Nylanderia (21 species)	*Nylanderia amia*
		Nylanderia aseta
		Nylanderia birmana
		Nylanderia bourbonica
		Nylanderia flavipes
		Nylanderia formosae
		Nylanderia gulinensis
		Nylanderia indica
		Nylanderia integera
		Nylanderia kraepelini
		Nylanderia opisopthalmia
		Nylanderia otome
		Nylanderia paraflavipas
		Nylanderia picta
		Nylanderia pieli
		Nylanderia ryukyuensis
		Nylanderia sharpii
		Nylanderia teranishii
		Nylanderia vividula
		Nylanderia yerburyi
		Nylonderia towori
	Oecophylla (1 species)	*Oecophylta smaragdina*
	Paraparatrechina (3 species)	*Paraparatrechina guanyin*
		Paraparatrechina kongming
		Paraparatrechina sauteri

	Paratrechina (1 species)	*Paratrechina longicornis*
	Plagiolepis (9 species)	*Plagiolepis alluaudi*
		Plagiolepis cardiocarenis
		Plagiolepis demangei
		Plagiolepis exigua
		Plagiolepis jerdonii
		Plagiolepis longwang
		Plagiolepis manczshurica
		Plagiolepis pallescens
		Plagiolepis pygmaea
	Polyergus (2 species)	*Polyergus rufescens*
		Polyergus samurai
	Polyrhachis (44 species)	*Polyrhachis armata*
		Polyrhachis bakana
		Polyrhachis bicolor
		Polyrhachis bihamata
		Polyrhachis brericorpa
		Polyrhachis conzera
		Polyrhachis cornihumera
		Polyrhachis cyphonota
		Polyrhachis debilis
		Polyrhachis demangei
		Polyrhachis dentihumera
		Polyrhachis dives
		Polyrhachis euthiacaena
		Polyrhachis furcata
		Polyrhachis halidayi
		Polyrhachis hippomanes
		Polyrhachis illaudata
		Polyrhachis jianghuaensis
		Polyrhachis laevigata
		Polyrhachis lamellidens
		Polyrhachis latona
		Polyrhachis lucidula
		Polyrhachis moesta
		Polyrhachis murina
		Polyrhachis nufipes
		Polyrhachis orbihumera
		Polyrhachis paracamponota
		Polyrhachis proxima
		Polyrhachis pubescens
		Polyrhachis punctillata
		Polyrhachis rastellata

		Polyrhachis rotoccipita
		Polyrhachis rubigastrica
		Polyrhachis schang
		Polyrhachis shixingensis
		Polyrhachis striata
		Polyrhachis subpilosa
		Polyrhachis thompsoni
		Polyrhachis thrinax
		Polyrhachis tibialis
		Polyrhachis tigilans
		Polyrhachis tschu
		Polyrhachis tyrannic
		Polyrhachis wolfi Forel
	Prenolepis (11 species)	*Prenolepis angularis*
		Prenolepis emmae
		Prenolepis flariabdominis
		Prenolepis longiventris
		Prenolepis maznocula
		Prenolepis melanogaster
		Prenolepis naoroji
		Prenolepis nigriflagella
		Prenolepis seplemdenta
		Prenolepis sphingthoraxa
		Prenolepis umbra
	Proformica (14 species)	*Proformica buddhaensis*
		Proformica coriacea
		Proformica dolichocephala
		Proformica epinotalis
		Proformica flarosetosa
		Proformica jacoti
		Proformica kascabi
		Proformica korbi
		Proformica mongolica
		Proformica nasuta
		Proformica nitida
		Proformica pilosiseapa
		Proformica splendida
		Proformicea striaticeps
	Pseudolasius (12 species)	*Pseudolasius bidenticlypeus*
		Pseudolasius binghami
		Pseudolasius cibdelus
		Pseudolasius emeryi
		Pseudolasius familiaris

		Pseudolasius hummeli
		Pseudolasius longiscapus
		Pseudolasius risii
		Pseudolasius salvazai
		Pseudolasius sauteri
		Pseudolasius silvestrii
		Pseudolasius similus
	Rossomyrmex (1species)	*Rossomyrmex quandratinodum*
Myrmicina (Total 41 genera, 392 species)	*Acanthomyrmex* (3 species)	*Acanthomyrmex crassispinus*
		Acanthomyrmer glabfemoralis
		Acanthomyrmex luciolae
	Anillomyrma (1 species)	*Anillomyrmez decamera subsp. continentis*
	Aphaenogaster (25 species)	*Aphaenogaster angulata*
		Aphaenogaster beccari
		Aphaenogaster caeciliae
		Aphaenogaster concolor
		Aphaenogaster ezasperata
		Aphaenogaster famelica
		Aphaenogaster feae
		Aphaenogaster geei
		Aphaenogaster hunanensis
		Aphaenogaster incurviclypea
		Aphaenogaster japomica
		Aphaenogaster lepida
		Aphaenogaster longiceps
		Aphaenogaster polyodonta
		Aphaenogaster pumilopuncta
		Aphaenogaster rothneyi
		Aphaenogaster schurri
		Aphaenogaster smythiesii
		Aphaenogaster suberaperata
		Aphaenogaster subterranea
		Aphaenogaster takahashii
		Aphaenogaster tibetana
		Aphaenogaster tipuna
		Aphaenogaster tokarainsulana
		Aphaenogaster weigoldi
	Atopomyrmex (1 species)	*Atopomyrmex srilankensis*
	Calyptomyrmex (1 species)	*Calyptomyrmer wittmeri*
	Cardiocondyla (8 species)	*Cardiocondyla elegans*
		Cardiocondyla insutura
		Cardiocondyla nigra

		Cardiocondyla nuda
		Cardiocondyla parvinoda
		Cardiocondyla stambuloffii
		Cardiocondyla wroughtoni var. obscurior
		Cardiocondyla wroughtonii
	Carebara (1 species)	*Carebara lignata*
	Cataulacus (1 species)	*Cataulacus granulatus*
	Crematogaster (39 species)	*Crematogaster aitkenii*
		Crematogaster anthracina
		Crematogaster artifex
		Crematogaster biroi
		Crematogaster bison
		Crematogaster brunnea subsp. ruginota
		Crematogaster brunnea var. nicevillei
		Crematogaster chungi
		Crematogaster contemta
		Crematogaster dohrni
		Crematogaster dohrni fabricans
		Crematogaster dohrni subsp. kiangsiensis
		Crematogaster ebenina
		Crematogaster egidyi
		Crematogaster ferrarii
		Crematogaster hogsomi
		Crematogaster jehovae
		Crematogaster macaoensis
		Crematogaster matsumurai
		Crematogaster millardi
		Crematogaster osakensis
		Crematogaster pia
		Crematogaster pia var. taivanae
		Crematogaster politula
		Crematogaster popohana
		Crematogaster popohana subsp. amia
		Crematogaster rogenhoferi
		Crematogaster ronganensis
		Crematogaster rothneyi
		Crematogaster sagei
		Crematogaster schimmeri

		Crematogaster subdentata
		Crematogaster subnuda
		Crematogaster subnuda formosae
		Crematogaster travancorensis
		Crematogaster treubi
		Crematogaster vagula
		Crematogaster wroughtomii
		Crematogaster zoceensis
	Dacatria (1 species)	*Dacatria templaris*
	Dilobocondyla (1 species)	*Dilobocondyla fouqueti*
	Eurhopalothrix (1 species)	*Eurhopalothrix procera*
	Gauromyrmex (1 species)	*Gauromyrmez acanthina*
	Kartidris (5 species)	*Kartidris ashima*
		Kartidris fujianensis
		Kartidris galos
		Kartidris nyos
		Kartidris sparsipila
	Leptothorax (14 species)	*Leptothorax acervorum*
		Leptothorax argentipes
		Leptothorax brevispinus
		Leptothorax confucii
		Leptothorax congruus var. wui
		Leptothorax eburneipes
		Leptothorax fultonii
		Leptothorax galeatus
		Leptothorax muscorum
		Leptothorax opaciabdomin
		Leptothorax reduncus
		Leptothorax reticulatus
		Leptothorax spinosior
		Leptothorax taivanensis
	Lophomyrmex (4 species)	*Lophomyrmex bedoti*
		Lophomyrmex birmanus
		Lophomyrmex quadrispinosus
		Lophomyrmex quadrispinosus subsp. taivanae
	Mayriella (1 species)	*Mayriella transfuga*
	Meranoplus (4 species)	*Meranoplus bicolor*
		Meranoplus bicolor var. fuscescens
		Meranoplus bicolor subsp.lucidus

		Meranoplus laeviventris
	Messor (10 species)	*Messor aciculatus*
		Messor aciculatus var.risianus
		Messor aralocaspius
		Messor aralocaspius subsp.infumatus
		Messor denticulatus
		Messor ezcursionis
		Messor inermis
		Messor striatellus
		Messor structor
		Messor valentinae
	Metapone (1 species)	*Metapone sauteri*
	Monomorium (21 species)	*Monomorium bimaculatum*
		Monomorium braunsi
		Monomorium chinense
		Monomorium concolor
		Monomorium destructor
		Monomorium floricola
		Monomorium hainanense
		Monomorium hiten
		Monomorium imperum
		Monomorium intrudens
		Monomorium intrudens subsp. pieli
		Monomorium latinode
		Monomorium latinodoides
		Monomorium mayri
		Monomorium monomorium
		Monomorium orientale
		Monomorium pharaonis
		Monomorium punctipectoris
		Monomorium sechellense
		Monomorium subopacum
		Monomorium triviale
	Myrmecina (7 species)	*Myrmecina graminicola*
		Myrmecina graminicola subsp.sinensis
		Myrmecina guangziensis
		Myrmecina sauteri
		Myrmecina striata
		Myrmecina strigis
		Myrmecina taiwana

	Myrmica (39 species)	*Myrmica angulata*
		Myrmica angulinodis
		Myrmica arisana
		Myrmica cachmiriensis
		Myrmica chinensis
		Myrmica draco
		Myrmica formosae
		Myrmica gallienii
		Myrmica helleri
		Myrmica inezae
		Myrmica jessensis
		Myrmica koreana
		Myrmica kozlovi
		Myrmica kozlovi subsp.mekongi
		Myrmica kozlovi subsp.subalpina
		Myrmica kozlovi subsp.subbrevispinosa
		Myrmica lobicornis
		Myrmica margaritae
		Myrmica mirabile
		Myrmica ritae
		Myrmica rubra
		Myrmica ruginodis
		Myrmica ruginodis var.khamensis
		Myrmica rugosa
		Myrmica saposhnikovi
		Myrmica scabrinodis
		Myrmica schenki
		Myrmica serica
		Myrmica sinensis
		Myrmica sinica
		Myrmica smythiesii
		Myrmica smythiesii subsp.exigua
		Myrmica stangeana
		Myrmica sulcinodis
		Myrmica taibaiensis
		Myrmica tibetana
		Myrmica urbanii
		Myrmica vandeli
		Myrmica wesmeali

	Myrmicaria (1 species)	*Myrmicaria brunnea*
	Oligomyrmex (18 species)	*Oligomyrmez acutispinus*
		Oligomvrmex amius
		Oligomyrmer altinodus
		Oligomyrmer reticapitus
		Oligomyrmer sauteri
		Oligomyrmer striatus
		Oligomyrmer taiponicus
		Oligomyrmex bihornatus
		Oligomyrmex capreolus
		Oligomyrmex curvispinus
		Oligomyrmex hunanensis
		Oligomyrmex jiangxiensis
		Oligomyrmex polyphemus
		Oligomyrmex rectidorsus
		Oligomyrmex wheeleri
		Oligomyrmez lusciosus
		Oligomyrmez obtusidentus
		Oligomyrmez pseudolusciosus
	Perissomyrmex (3 species)	*Perissomyrmex bidentatus*
		Perissomyrmex fissus
		Perissomyrmex guizhouensis
	Pheidole (48 species)	*Pheidole allani*
		Pheidole amia
		Pheidole aphrasta
		Pheidole binghamii
		Pheidole capellinii
		Pheidole constanciae
		Pheidole ernsti
		Pheidole feae
		Pheidole fervens
		Pheidole fervens soror
		Pheidole fervens var.dolenda
		Pheidole fervida
		Pheidole flaveria
		Pheidole funkikoensis
		Pheidole hongkongensis
		Pheidole indica
		Pheidole indosinensis
		Pheidole jucunda
		Pheidole longiscapus
		Pheidole malinsii
		Pheidole megacephala

		Pheidole meihuashanensis
		Pheidole multidens
		Pheidole nietneri
		Pheidole noda
		Pheidole noda var. flebilis
		Pheidole noda var.formosensis
		Pheidole nodgii var.zoceana
		Pheidole nodifera
		Pheidole ocellata
		Pheidole pieli
		Pheidole rinae subsp. incensa
		Pheidole rinae tipuna
		Pheidole roberti
		Pheidole sagei
		Pheidole sauteri
		Pheidole selathoraz
		Pheidole sinica
		Pheidole smythiesii
		Pheidole spathifera
		Pheidole sulcaticeps
		Pheidole taipoana
		Pheidole taivanensis
		Pheidole teneriffana
		Pheidole tsailuni
		Pheidole watsoni
		Pheidole yeensis
		Pheidole zhoushanensis
	Pheidologeton (12 species)	*Pheidologeton affinis*
		Pheidologeton dentivirus
		Pheidologeton diversus
		Pheidologeton diversus subsp. laotinus
		Pheidologeton draco
		Pheidologeton fictus
		Pheidologeton latinodus
		Pheidologeton melasolenus
		Pheidologeton nanningensis
		Pheidologeton trechiderus
		Pheidologeton vespillo
		Pheidologeton yanoi
	Pristomyrmex (4 species)	*Pristomyrmex brevispinosus*
		Pristomyrmer formosae
		Pristomyrmer hamatus

		Pristomyrmex pungens
	Pyramica (26 species)	*Pyramica ailaoshana*
		Pyramica benten
		Pyramica canina
		Pyramica dayui
		Pyramica dohertyi
		Pyramica elegantula
		Pyramica emeswangi
		Pyramica formosa
		Pyramica formosimonticola
		Pyramica heramera
		Pyramica hirashimai
		Pyramica japonica
		Pyramica kichijo
		Pyramica lachesis
		Pyramica leptothrix
		Pyramica mazu
		Pyramica membranifera
		Pyramica mitis
		Pyramica mutica
		Pyramica nongba
		Pyramica sauteri
		Pyramica sinensis
		Pyramica takasago
		Pyramica tisiphone
		Pyramica wilsoni
		Pyramica yangi
	Recurvidris (3 species)	*Recurvidris glabriceps*
		Recurvidris nuwa
		Recurvidris recurvispinosa
	Rhopalomastix (1 species)	*Rhopalomastix Forel*
	Rhoptromyrmex (1 species)	*Rhoptromyrmer wroughtonii*
	Rotastruma (1 species)	*Rotastruma stenoceps*
	Solenopsis (8 species)	*Solenopsis geminata*
		Solenopsis indagatrix
		Solenopsis jacoti
		Solenopsis jacoti pekingensis
		Solenopsis saevissima
		Solenopsis soochowensis
		Solenopsis tipuna
		Solenopsis wagneri
	Stenamma (1 species)	*Stenamma orustoni*
	Strongylognathus (5 species)	*Strongylognathus chelifer*

		Strongylognathus karawajewi
		Strongylognathus koreanus
		Strongylognathus potanini
		Strongylognathus tylonum
	Strumigenys (19 species)	*Strumigenys chuchihensis*
		Strumigenys emmae
		Strumigenys exilirhina
		Strumigenys formosensis
		Strumigenys hispida
		Strumigenys jiangxiensis
		Strumigenys konteiensis
		Strumigenys lacunose
		Strumigenys lewisi
		Strumigenys lichiaensis
		Strumigenys liukueiensis
		Strumigenys minutula
		Strumigenys nanzanensis
		Strumigenys orchidensis
		Strumigenys pilosa
		Strumigenys rallarhina
		Strumigenys solifontis
		Strumigenys strygar
		Strumigenys trada
	Tetramorium (46 species)	*Tetramorium amium*
		Tetramorium aptum
		Tetramorium bicarinatum
		Tetramorium caespitum
		Tetramorium caespitum var. pallidum
		Tetramorium ciliatum
		Tetramorium crepum
		Tetramorium cuneinode
		Tetramorium cyclolobium
		Tetramorium dunhuangense
		Tetramorium feror
		Tetramorium flavum
		Tetramorium forte
		Tetramorium guangriense
		Tetramorium indicum
		Tetramorium indosinense
		Tetramorium inerme
		Tetramorium inglebyi
		Tetramorium insolens

		Tetramorium kheperrum
		Tetramorium khnum
		Tetramorium kraepelini
		Tetramorium lanuginosum
		Tetramorium laparum
		Tetramorium mai
		Tetramorium nipponense
		Tetramorium nursei
		Tetramorium obtusidens
		Tetramorium ochrothorax
		Tetramorium pacificum
		Tetramorium parvispinum
		Tetramorium pilosum
		Tetramorium repletum
		Tetramorium rizangense
		Tetramorium schneideri
		Tetramorium shensiense
		Tetramorium simillimum
		Tetramorium smithi
		Tetramorium striabdomen
		Tetramorium tonganum
		Tetramorium tsushimae
		Tetramorium turcomanicum
		Tetramorium undatium
		Tetramorium walshi
		Tetramorium yerburyi
		Tetramorium yulongense
	Vollenhovia (4 species)	*Vollenhovia emeryi*
		Vollenhovia lucimandibula
		Vollenhovia pyrrhoria
		Vollenhovia satoi
	Vombisidris (1 species)	*Vombisidris umbrabdomina*
Ponerinae (Total 14 genera, 117 species)	*Anochetus* (5 species)	*Anochetus graeffei*
		Anochetus risii
		Anochetus subcoecus
		Anochetus taiwaniensis
		Anochetus yunnanensis
	Centromyrmex (1 species)	*Centromyrmex feae*
	Cryptopone (8 species)	*Cryptopone butteli*
		Cryptopone gigas
		Cryptopone jinxiuensis
		Cryptopone pseudogigas
		Cryptopone recticlypea

		Cryptopone sauteri
		Cryptopone sinensis
		Cryptopone taivanae
	Diacamma (3 species)	*Diacamma pallidum*
		Diacamma rugosum
		Diacamma viridipurpureum
	Emeryopone (1 species)	*Emeryopone melaina*
	Harpegnathos (2 species)	*Harpegnathos saltator*
		Harpegnathos venator
	Hypoponera (13 species)	*Hypoponera bebpin*
		Hypoponera biroi
		Hypoponera confinis
		Hypoponera cwaluwenburgi
		Hypoponera excoecata
		Hypoponera gleadowi
		Hypoponera nippona
		Hypoponera opacicebs
		Hypoponera sauteri
		Hypoponera schauinslandi
		Hypoponera silvestrii
		Hypoponerabondroit
		Hypoponeratruncata
	Leptogenys (23 species)	*Leptogenys binghamii*
		Leptogenys birmana
		Leptogenys chinensis
		Leptogenys confucii
		Leptogenys crassicornis
		Leptogenys diminuta
		Leptogenys ergatogyna
		Leptogenys hezhouensis
		Leptogenys huapingensis
		Leptogenys kitteli
		Leptogenys kraepelini
		Leptogenys laeviterga
		Leptogenys laozii
		Leptogenys lucidula
		Leptogenys mengzii
		Leptogenys pangui
		Leptogenys peuqueti
		Leptogenys punctiventris
		Leptogenys rufida
		Leptogenys strena
		Leptogenys sulcifrons

		Leptogenys yerburyi
		Leptogenys zhuangzii
	Myopias (2 species)	*Myobias conicara*
		Myopias nops
	Odontomachus (8 species)	*Odontomachus circulus*
		Odontomachus fulgidus
		Odontomachus granatus
		Odontomachus haematodus
		Odontomachus monticola
		Odontomachus rirosus
		Odontomachus rizangensis
		Odontomachus tensus
	Odontoponera (1 species)	*Odontoponera transversa*
	Pachycondyla (24 species)	*Pachycondyla amblyops*
		Pachycondyla annamita
		Pachycondyla astuta
		Pachycondyla bilosior
		Pachycondyla bispinosa
		Pachycondyla brevidorsa
		Pachycondyla caffraria
		Pachycondyla cavimaculata
		Pachycondyla chinensis
		Pachycondyla darwini
		Pachycondyla javana
		Pachycondyla leeuuenhoeki
		Pachycondyla lobocarena
		Pachycondyla luteipes
		Pachycondyla melanaria
		Pachycondyla nigrita
		Pachycondyla rubiginosa
		Pachycondyla rufipes
		Pachycondyla sauteri
		Pachycondyla schaeferi
		Pachycondyla sharpi
		Pachycondyla stigma
		Pachycondyla tianzun
		Pachycondyla tonkina
	Platythyrea (1 species)	*Platythyrea clypeata*
	Ponera (25 species)	*Ponera alisana*
		Ponera baka
		Ponera bawana
		Ponera chiponensis
		Ponera diodonta

		Ponera grandis
		Ponera guangxiensis
		Ponera hubeiensis
		Ponera iabonica
		Ponera lonelina
		Ponera menglana
		Ponera nangongshana
		Ponera paedericera
		Ponera pentodontos
		Ponera pianmana
		Ponera rishen
		Ponera scabra
		Ponera shennong
		Ponera sinensis
		Ponera taiyangshen
		Ponera takaminei
		Ponera tamon
		Ponera tenuis
		Ponera xantha
		Ponera yuhuang
Proceratiinae (Total 3 genera, 10 species)	*Discothyrea* (2 species)	*Discothyrea sauteri*
		Discothyrea yueshen
	Probolomyrmex (2 species)	*Probolomyrmer longinodus*
		Probolomyrmex longiscapus
	Proceratium (6 species)	*Proceratium itoi*
		Proceratium japonicum
		Proceratium longigaster
		Proceratium longmenense
		Proceratium nujiangense
		Proceratium zhaoi
